# Waffle production: influence of batter ingredients on sticking of waffles at baking plates—Part II: effect of fat, leavening agent, and water

**DOI:** 10.1002/fsn3.425

**Published:** 2016-09-20

**Authors:** Regina Huber, Regine Schoenlechner

**Affiliations:** ^1^ Department of Food Sciences and TechnologyUniversity of Natural Resources and Life SciencesViennaAustria

**Keywords:** Fat, fresh egg waffle, leavening agents, sticking, waffle batter

## Abstract

Fresh egg waffles are continuously baked in tunnel baking ovens in industrial scale. Waffles that partly or fully stick to the baking plates cause significant product loss and increased costs. The aim of this study was, therefore, to investigate the effect of different recipe ingredients on the sticking behavior of waffles. In this second part, ingredients investigated were different leavening agents (sodium acid pyrophosphate, ammonium bicarbonate, magnesium hydroxide carbonate, or monocalcium phosphate), different fat sources (rapeseed oil, cocos fat, butter, or margarine), and different water sources (tap water 12°dH and distilled water). Within the different types of fats, solid fats with high amount of short‐chain fatty acids (cocos fat or butter) decreased the number of sticking waffles compared to liquid oils (rapeseed oil). Regarding leavening agents, magnesium hydroxide carbonate and ammonium bicarbonate were superior to sodium acid pyrophosphate or monocalcium phosphate. Between the two water sources, effects were small.

## 1 Introduction

Fresh egg waffles (subsequently called waffles) are made from high contents of eggs, sugar, and fat. They have a raised, cake‐like texture and are baked in waffle irons. An industrial waffle oven produces up to 40,000 waffles per hour and only 1% of sticking waffles can make up to 72 kg waste per day (Tiefenbacher, 2009). Thus, sticking of waffles on baking plates must be prevented, but the reasons for sticking are not fully understood yet. Influencing factors on sticking of waffles are recipe ingredients, the baking process, baking plate material, release agent, and cleaning methods.

In this study the following influences of some batter ingredients were investigated: type of fat, leavening agents, and water hardness.

Fat and oils in waffle batters lead to softer texture of the waffle. Besides, fat provides a release film during baking. Disadvantageous effects are residues of fat waste on baking plates. Depending on the chain length and number of unsaturated fatty acids, fats show different stability. The more double bonds in fatty acids, the more thermal instable they are due to autoxidation. For waffles, stable fats with little amount of unsaturated fatty acids and iodine values below 85 should be used to minimize thermal fat degradation and increase shelf life. Required fat amount depends on the recipe and other batter ingredients and baking plate coating. Usual fat amounts of waffle batters are 18–29% (Tiefenbacher, 2009). According to Pareyt et al. (2009), fat might retard chemical leavening action and affect swelling of the cells during baking of cookies. Surface became uneven with decreasing fat levels. Lecithin (E322) is often used in waffle batters, where it acts as release improver and is therefore also an important ingredient in release agents. Additionally, lecithin improves the color distribution on the waffle surface. In waffles, liquid or powdered lecithin is usually used from 0.1% to 1.5% of flour (Tiefenbacher, Haas, & Haas, 1991).

Leavening agents are chemicals like sodium bicarbonate, sodium acid pyrophosphate, ammonium bicarbonate, magnesium hydroxide carbonate, and monocalcium phosphate, which lead to better steam release, softer, more porous, and lighter waffles (Tiefenbacher et al., 1991). Sodium bicarbonate (E500ii) increases the pH, which leads to more intensive browning and increases alkaline taste (Hesso et al., 2015). Ammonium bicarbonate (E503i) increases the pH as well, but pore size is more homogeneous, which makes the waffle softer and increases volume. It has a strong characteristic flavor and increases waste on baking plates. Ammonium bicarbonate is added at a maximum of 0.1% (Tiefenbacher, 2009). Sodium pyrophosphate (E450i) reacts with sodium bicarbonate and should be used in the ratio 1:0.73. Too much sodium pyrophosphate gives a metallic taste. Monocalcium phosphate (E341i) has a very fast leavening reaction compared to the other leavening agents and is used usually for American breakfast waffles (Miller, 2016). Magnesium hydroxide carbonate (E504) improves waffle release and can protect water from moisture migration.

Water temperature should be constant for batter preparation at about 20–30°C, because the warmer the water, the thicker gets the batter. Higher water hardness influences waffle texture and may require a reduced amount of leavening agents. But hard water increases baking plate residues. Soft water provides smoother waffle texture, which decreases waffle stability. Stable waffles can be taken off easier (Tiefenbacher, 2009). According to a study of Liu, Christian, Zhang, and Fryer (2002), water content influenced the adhesive strength of fouling from tomato paste. The adhesive strength of the tomato deposit could be reduced by hydration.

The objective of this study was to analyze the influence of batter ingredients on the quality (stability) and sticking behavior of waffles. Analyzed ingredients for this part of the study were: (1) the effect of different leavening agents (sodium acid pyrophosphate, ammonium bicarbonate, magnesium hydroxide carbonate, or monocalcium phosphate), (2) the effect of different fat sources (rapeseed oil, cocos fat, butter, or margarine), and (3) the effect of water source (tap water 12°dH and distilled water) on the quality (stability) of the waffle and on its release from the baking plates. All waffle trials were performed on pilot scale using equipment that is usually used in industrial baking.

## 2 Materials and Methods

### 2.1 Materials

The following flour and starch ingredients were used for this study: wheat flour W480 (type “Allerfeinst,” Good Mills GmbH, Schwechat, Austria; protein content 11% dm, starch content 72% dm), eggs pasteurized (Landgold Fresh GmbH, Wien, Austria), tap water (12°dH, Leobendorf, Austria), sorbitol syrup (Sorbitex Ltd., Cincinnati, OH, USA), glycerin syrup (type 1.23, Neuber's Enkel, Vienna, Austria), sugar (type crystal, Agrana GmbH, Tulln, Austria), skimmed milk powder (DMK GmbH, Zeven, Germany), citric acid (Anna Gold GmbH, Vienna, Austria), the emulsifiers monoglyceride (“Colco mono,” Co. Aromatic, Stockholm, Sweden), lecithin (from soy, liquid, “Lecisoya” F60IP, Werba GmbH, Vienna, Austria), the leavening agents ammonium bicarbonate (Anna Gold, Vienna, Austria), sodium acid pyrophosphate SAPP40 (Levall, Brenntag, Antwerp, Belgium), magnesium hydroxide carbonate (Neuber's Enkel, Vienna, Austria), monocalcium phosphate (Neuber's Enkel, Vienna, Austria), and the fats rapeseed oil (100% refined, Ölwert GmbH, Langenlois, Austria), margarine (Rama Original, vegetable fat, Unilever Austria, Vienna, Austria), butter (Spar, Salzburg, Austria), cocos fat partly hardened (Ceres, Wels, Austria). “Bandex” (Co. Aromatic, Stockholm, Sweden) was used as release agent applied on the baking plates. All raw materials were stored in cool place (8–10°C) and taken out from the cooling room 15 min prior to batter preparation.

### 2.2 Experimental design

The principal waffle recipe and its variations are given in Table 1. There is no standard recipe for fresh egg waffles. The recipe used for this study was based on a typical industrial scale waffle recipe as used and applied by CFT Haas Convenience Food Equipment GmbH, Leobendorf, Austria. The used ingredients and their amounts were standardized in pretrials (results not presented here).

**Table 1:** Experimental design—principal recipe of waffles investigated

Ingredients	Mixing sequence	Principle recipe(%)
Wheat flour W480	3	22.40
Water 12°dH^c^	1	7.96
Egg, fresh	1	24.89
Sorbitol syrup	1	4.98
Glycerin syrup	1	2.49
Sugar crystal	2	15.93
Skimmed milk powder	2	1.00
Sodium bicarbonate	2	0.29
Citric acid	2	0.05
SAPP40^a^	3	0.4
Monoglyceride colco M	3	0.25
Lecithin liquid	4	0.50
Rapeseed oil^b^	4	18.86
Sum	‐	100.00

a To investigate the effect of leavening agent, the full amount of SAPP40 was replaced by ammonium bicarbonate, magnesium hydroxide carbonate, or monocalcium phosphate.

b To investigate the effect of different fat sources, the full amount of rapeseed oil was replaced by cocos fat, butter, or margarine.

c To investigate the effect of water, the full amount of tap water (12°dH) was replaced by distilled water.

In order to study the effect of leavening agent, the full amount of SAPP40 was replaced by ammonium bicarbonate, magnesium hydroxide carbonate, or monocalcium phosphate. The effect of different fat sources was studied by replacing the full amount of rapeseed oil by cocos fat, butter, or margarine, the effect of water was studied by replacing the full amount of tap water (12°dH) by distilled water (see Table 1).

All these waffles were produced in the way industrial waffles are produced, using the same batter mixing machines and baking plates (see section 2.3). From each recipe, 30 waffles were baked and evaluated in order to obtain reliable results.

### 2.3 Preparation of waffle batter

Preparation of batter was performed using a dissolver stirrer (IKA‐Werke GmbH & Co., KG, Staufen, Germany) at medium speed of 800 rpm according to following common mixing sequence steps for waffles: Step 1—water and liquids (eggs, sorbitol, glycerin), 1 min; Step 2—water‐soluble powders (sugar, skimmed milk powder, citric acid, first leavening agent), 3–4 min; Step 3—flour, starches, and pastes (flour, starch, monoglyceride, eventually second leavening agent), 3–4 min; Step 4—fat and lecithin prewarmed to 40°C, 1–2 min. The mixing of the waffle batter in this sequence is important to avoid too high shearing of the batter, which would result in increased gluten development, which is not desired for waffle production. The mixed batter was allowed to rest for 15–30 min prior to baking. Batter preparation was kept constant for all recipes. All batters were monitored for pH value, temperature, density, and viscosity in order to detect eventual influences of the varied recipe parameters.

### 2.4 Baking process for waffle production

Before baking, the baking tong “Turtle” (ductile iron baking plates, FHW Haas, Leobendorf, Austria) was preheated up to 140°C (bottom) and 145°C (top baking plate). Temperature of baking plates was monitored during the trials and found to be stable within a range of ±5°C. Before first batter disposition, a constant amount of the release agent (Bandex) was spread on both baking plates. For baking, a constant amount of 15 ml batter was deposited on the bottom baking plate, the baking plate was closed, and the baking process started immediately (110 s). The tong was then opened and the waffle removed from the baking tong using a self‐prepared vertical needle take‐off (CFT Haas Convenience Food Equipment GmbH, Leobendorf, Austria), which is the usual process in industrial scale waffle production.

After a cooling period of 30 min (time for moisture equilibration within the waffle), the waffle was characterized for weight, color, color spots, moisture content, and water activity as described in the following section. Additionally, after baking of all 30 waffles and complete cooling, the baking plate was characterized visually by a microscope and for surface roughness by a surface roughness analyzer (Hommel Tester T100, Hommel Seitz, Viernheim, Germany) to ensure that the baking plate surface has not changed by the baking or cleaning process, which would have an influence on sticking behavior of the waffle. During this study, surface roughness was found to be constant. Between different recipes cleaning was carried out by dry ice (frozen CO_2_ pellets) in order to provide the same starting conditions for all recipes. In case a recipe produced many sticking waffles, an in‐between cleaning step had to be done by brushing and applying new release agent. In the Results section, information is given for which recipes this step had to be applied.

### 2.5 Characterization of batter parameters (pH value, temperature, batter density, and batter viscosity)

The methods for determination of the pH value, temperature, batter density, and batter viscosity are described in detail in the first part of this study (Huber & Schoenlechner, 2016). All these measurements were performed in triplicate and given as mean value.

### 2.6 Characterization of waffles (baking loss, moisture content, water activity, color, and color distribution)

The method for measuring the baking loss, the moisture content, the water activity, the color, and the color distribution is the same procedure as in the first part of this study and described in detail previously (Huber & Schoenlechner, 2016). The baking loss was calculated from 30 measured waffles, as well as the moisture content, water activity, and color.

### 2.7 Determination of adhesion force and number of sticking waffles

**Figure 1:**
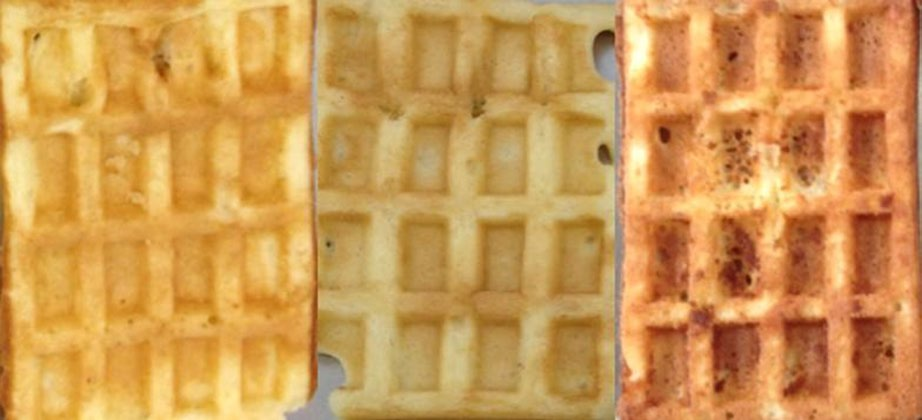
Waffles baked with palm fat (left), magnesium hydroxide carbonate (middle), and water of 12°dH (right)

On industrial scale, usually waffles are taken off from the baking plates vertically by needles. In order to best simulate this process, a so‐called needle take‐off was simulated by an in‐house construction for pilot scale production, which allowed measuring the force required to take off the waffle (see Fig. 1). After opening the baking tong, the arm with the needles was moved above the waffle, the needles were put into the waffle always at the same position, the scale was tared, and then the needles were pulled up vertically by pneumatic force. The pneumatic pressure and the angle of the take‐off arm was set constant and proofed by a barometer. A hanging scale (HS 300± 0.1 g, Co. Dipse, Oldenburg, Germany) recorded the weight required to take‐off the waffle from the baking tong, which was used to calculate the adhesion force by the following formula:

$$\text{Adhesion force}\left[N=kg\times m/s^{2}\right]=\text{Waffle take‐off weight}\left[g\right]/1000\times 9.81\left[m/s^{2}\right].$$

The weight of the waffles was included in this calculation, but all waffles were baked from exactly the same weight (weight of waffles after baking were controlled). Depending on the intensity of adhesion, the weight of the taken‐off waffle increased.

Number of sticking waffles was counted for all 30 waffles produced for one recipe and given as %. Torn waffles (not or only half taken off) were considered as sticking waffles.

### 2.8 Statistical evaluation

Results of waffles were evaluated statistically using Statgraphics Centurion XVI, Statpoint Technologies, Inc., Warrenton, VA, USA) by one‐factor analysis of variance (ANOVA) and Spearman's rank correlation between each pair of variables. A *p* < .05 was considered as significant. Mean values were compared by Fisher's least significant difference (LSD) procedure.

## 3 Results and Discussion

All batter parameters determined are summarized in Table 2, and waffle parameters in Tables 3 and 4. All correlation values are presented in Table 5.

**Table 2:** Batter characterization: pH, temperature, density, and viscosity

Batter characterization	pH*n* = 3	Temperature(°C)*n* = 3	Density(g/L)*n* = 3	Viscosity(s)*n* = 3
Effect of leavening agents				
Ammonium	7.21 ± 0.01^d^	23.23 ± 0.06^a^	96.25 ± 2.69^c^	32 ± 1^a^
Magnesium	6.84 ± 0.02^c^	26.67 ± 0.31^d^	94.02 ± 1.51^bc^	38 ± 0^bc^
MCP	6.44 ± 0.01^b^	24.33 ± 0.21^b^	88.32 ± 0.43^a^	240 ± 0^d^
SAPP40‐Sodium ISTD	6.36 ± 0.01^a^	26.60 ± 0.20^c^	92.47 ± 0.42^b^	120 ± 4^c^
*p*‐value	.0000	.0000	.0017	.0000
Effect of fat and lecithin				
Margarine 3	6.31 ± 0.01^a^	28.17 ± 0.15^d^	90.17 ± 1.24^a^	45 ± 1^a^
Cocos fat 4	6.41 ± 0.02^c^	20.03 ± 0.06^a^	95.95 ± 0.63^cd^	60 ± 2^b^
Butter 5	6.29 ± 0.01^a^	23.40 ± 0.26^b^	94.66 ± 0.78^c^	111 ± 8^c^
Rapseed oil ISTD 1	6.36 ± 0.01^b^	26.60 ± 0.20^c^	92.47 ± 0.42^b^	120 ± 4^cd^
*p*‐value	.0000	.0000	.0000	.0000
Effect of water hardness				
Water distilled	6.38 ± 0.03^a^	21.17 ± 0.12^a^	93.79 ± 0.72^a^	149 ± 3^b^
Water 12°dH ISTD	6.36 ± 0.01^a^	26.60 ± 0.20^b^	92.47 ± 0.42^a^	120 ± 4^a^
*p*‐value	.3276	.0000	.0520	.0010

Note

Different superscript letters in the same column denote significant differences (p>0.05).

**Table 3:** Waffle characterization: color values (L*‐, a*‐, b*‐values)

	L*n* = 30	a*n* = 30	b*n* = 30
Effect of leavening agents			
Ammonium	62.22 ± 5.01^b^	8.67 ± 2.06^b^	33.01 ± 1.27^b^
Magnesium	68.20 ± 3.06^c^	7.04 ± 1.55^a^	34.50 ± 1.62^c^
MCP	70.92 ± 3.26^d^	6.31 ± 2.31^a^	32.37 ± 2.34^b^
SAPP40‐Sodium ISTD	46.31 ± 6.61^a^	16.58 ± 2.93^c^	29.94 ± 2.56^a^
*p*‐value	.0000	.0000	.0000
Effect of fat and lecithin			
Margarine	63.76 ± 5.26^c^	11.92 ± 2.96^b^	34.75 ± 2.08^c^
Cocos fat	67.24 ± 4.25^c^	9.10 ± 3.13^a^	32.40 ± 1.98^b^
Butter	68.02 ± 2.86^b^	10.02 ± 2.05^a^	35.26 ± 1.64^c^
Rapseed oil ISTD	46.31 ± 6.61^a^	16.58 ± 2.93^c^	29.94 ± 2.56^a^
*p*‐value	.0000	.0000	.0000
Effect of water hardness			
Water distilled	62.30 ± 4.12^b^	11.79 ± 2.29^b^	33.44 ± 1.82^b^
Water 12°dH ISTD	46.31 ± 6.61^a^	16.58 ± 2.93^a^	29.94 ± 2.56^a^
*p*‐value	.0000	.0000	.0000

Note

Different superscript letters in the same column denote significant differences (p>0.05).

**Table 4:** Waffle characterization: baking loss, moisture content, aw‐value, take‐off force, and sticking of waffles

	Baking loss(%)*n* = 30	Moisture(%)*n* = 3	aw*n* = 3	Force(N)*n* = 30	Sticking(%)*n* = 30
Effect of leavening agents					
Ammonium	21.38 ± 3.27^a^	12.86 ± 0.55^a^	0.69 ± 0.01^a^	0.022 ± 0.004^a^	0.0^a^
Magnesium	16.51 ± 2.20^c^	17.89 ± 1.08^b^	0.76 ± 0.03^b^	0.014 ± 0.003^b^	0.0^b^
MCP	18.38 ± 1.62^b^	16.71 ± 0.83^b^	0.75 ± 0.02^b^	0.020 ± 0.000^ab^	6.7^b^
SAPP40‐Sodium ISTD	25.41 ± 3.31^d^	13.31 ± 0.94^a^	0.68 ± 0.03^a^	0.044 ± 0.015^c^	23.3^b^
*p*‐value	.0000	.0002	.0041	.0000	.0010
Effect of fat sources					
Margarine	22.32 ± 3.09^b^	15.91 ± 0.86^bc^	0.71 ± 0.03^a^	0.035 ± 0.008^b^	3.3^a^
Cocos fat	19.93 ± 2.39^a^	15.02 ± 0.93^ab^	0.72 ± 0.02^ab^	0.022 ± 0.002^a^	0.0^a^
Butter	20.08 ± 1.24^a^	17.53 ± 0.97^c^	0.75 ± 0.01^b^	0.021 ± 0.005^a^	0.0^a^
Rapseed oil ISTD	25.41 ± 3.31^c^	13.31 ± 0.94^a^	0.68 ± 0.03^a^	0.044 ± 0.015^c^	23.3^b^
*p*‐value	.0000	.0156	.0234	.0000	.0000
Effect of water hardness					
Water distilled	22.40 ± 2.14^a^	14.19 ± 1.29^a^	0.72 ± 0.02^a^	0.030 ± 0.007^a^	30.0^a^
Water 12°dH ISTD	25.41 ± 3.31^b^	13.31 ± 0.94^a^	0.68 ± 0.03^a^	0.044 ± 0.015^b^	23.3^a^
*p*‐value	.0001	.3946	.0936	.0000	.5670

Note

Different superscript letters in the same column denote significant differences (p>0.05).

**Table 5:** Correlation analyses of determined batter and waffle parameters

	Temp.	density	viscosity	moisture	aw‐value	L*	a*	b*	Baking loss	Sticking of waffles	Force
pH	−0.1214	0.1976	−0.2999	−0.1079	0.0605	0.3297	−0.6779^a^	−0.2357	−0.6828^a^	0.0001	−0.0242
Temperature		−0.705^a^	−0.28	0.2525	0.1862	0.0647	0.2153	0.3167	0.1519	0.0001	0.2333
Density			−0.3158	−0.1545	−0.2172	0.0311	−0.2455	−0.0257	−0.3657	0.0001	−0.3037
Viscosity				0.0174	0.0826	−0.2336	0.1891	−0.3024	0.3756	0.0001	0.3079
Moisture					0.8371^a^	0.4^a^	−0.1841	0.4706^a^	−0.1789	0.0001	−0.3838
aw						0.4656^a^	−0.3832	0.2415	−0.2483	0.0001	−0.6158^a^
L^a^							−0.8502^a^	0.1021	−0.7109^a^	−0.0113	−0.5956^a^
a*								0.1443^a^	0.745^a^	0.0130	0.6420^a^
b*									−0.1549^a^	−0.0783	−0.0875
Baking loss										0.0475	0.5558^a^
Sticking of waffles											−0.1679

a Significant correlation at α=0.05.

### 3.1 Effect of ingredients on batter parameters

Based on pretrials and experience by CFT Haas GmbH (Leobendorf, Austria), the control batter was composed in a way that the pH, density, and viscosity are within a recommended range for batters. Based on this recipe, the effect of varied ingredients was investigated and it was monitored if and in what range they changed these values.

The pH value of the batters was between 6.3 and 7.2. In the group of leavening agents, highest pH value was achieved with ammonium bicarbonate, lowest with SAPP40. Fats also caused significant differences in pH value, but differences were low. Water source had no effect on pH value. It is known that batter with higher pH values tend to increase the browning reaction of the batter, which can cause increased dough residues on the baking plate and subsequently increased number of sticking waffles. In industrial scale production (long‐term baking), this phenomenon can often be observed, usually by the use of higher amounts of ammonium bicarbonate or older eggs (CFT own experience).

Batter temperature ranged from 20 to 28°C, and did not significantly influence sticking behavior.

Density showed little variation, ranging from 88 to 96per 100 ml, but it was significantly correlated with sticking behavior. The higher the density, the lower was the number of sticking waffles. Higher batter density was found by addition of ammonium bicarbonate or magnesium hydroxide carbonate and by addition of cocos fat or butter. The leavening reaction of ammonium bicarbonate and magnesium hydroxide carbonate seems to be less intensive than the addition of SAPP40. Less gaseous chemical reactions seem to act after mixing. Viscosity showed higher variation from 32 to 149 s, yet it was not significantly correlated to the number of sticking waffles. Viscosity was lowest with ammonium bicarbonate or magnesium hydroxide carbonate and cocos fat or margarine, which is comparable to the relationship of density values.

Although these investigated parameters showed that principally all batter recipes were in a range that made them suitable for waffle production, it could be found that within the leavening agents, ammonium bicarbonate or magnesium hydroxide carbonate were somewhat superior. Within the fat sources, cocos fat, margarine, or butter seemed to better suitable for waffle production than rapeseed oil. This might be attributed to its physical parameters. Solid fats served better compared to liquid oils.

### 3.2 Effect of ingredients on general waffle quality

All investigated recipe parameters influenced significantly color, moisture content, water activity, and baking loss.

Color was significantly brighter after the addition of ammonium bicarbonate, which was unexpected, and after the addition of monocalcium phosphate. Also, fat source influenced color; after the addition of rapeseed oil, waffles were significantly darker, those with butter and cocos fat were brighter and less red. Distilled water resulted in brighter, less red, but more yellow waffles than tap water with higher hardness. Baking loss was affected by the different leavening agents in the following order: ammonium bicarbonate > monocalcium phosphate > magnesium hydroxide carbonate > SAPP40, but these phenomena could not be explained. Margarine and rapeseed oil caused higher baking loss compared to butter and cocos fat. It seemed that fats containing more short‐chain fatty acids retained water in the waffle better compared to fats containing higher amounts of long‐chain fatty acids.

Moisture content and aw‐value were higher in waffles with magnesium hydroxide carbonate and monocalcium phosphate and also fat source had significant effects on these parameters. Waffles with butter showed higher moisture content and aw‐value compared to the other fat sources (except margarine for moisture content and cocos fat for aw‐value). In contrast to the used fats and oils, butter contains much more water. Water hardness had no effects on batter moisture and its aw‐value. Only little research was done so far to investigate the effect of different fat sources or leavening agents on color of the resulting bakery products, in contrast to the effect of different flours. Their contribution to browning due to Maillard reactions has been investigated by several researchers (Miller, 2016; Vetter, 2003). Also, influence of water hardness is not well understood for bakery products, but it is well known that it can change the quality of distillates like Whiskey for example (Bringhurst & Brosnan, 2014). The phenomenon of changing waffle cohesion was observed during previous tests with wafers. Wafers produced with distilled water were far more crumbly brittle than wafers produced with water of 12°dH water hardness (CFT own experience).

Several correlations between color values, moisture content, water activity, and baking loss could be detected. Darker waffles (lower L‐value) was correlated with lower moisture content and lower aw‐value, which can be explained by the relation that from a darker and more browned waffle more steam is evaporated off. This was also expressed in an increased baking loss value (significant correlation of L‐value and baking loss).

### 3.3 Effect of ingredients on sticking behavior of waffles

The results of sticking behavior were determined by calculating the number of sticking waffles (% of 30 waffles) and measuring the take‐off force, which was recorded in order to have a tool to measure how easily the waffle could be removed from the baking plate or how much it adhered to it.

Take‐off force and number of sticking waffles were significantly influenced by the studied ingredients. SAPP40 showed highest take‐off force and higher number of sticking waffles compared to the other three leavening agents. Within the fat sources, cocos fat (see Fig. 1) and butter showed lowest take‐off force and number of sticking waffles. These waffles showed the best stability and thus performed better during production. For bakery products the use of solid fats at room temperature (including more saturated fatty acids) is generally recommended, because they are more heat stable on the one hand, but also have different flow behavior compared to oils that contain more unsaturated fatty acids. Solid fats like cocos fat or butter show more elastic and pseudoplastic properties in contrast to oils (Palav, 2016; Schünemann & Treu, 2009), so type of fat/oil not only influences release behavior, but has also effects on the baking characteristics, product structure (Hadnađev, Hadnađev, Milica, Slađana, & Veljko, 2015; Sciarini, Van Bockstaele, Nusantoro, Pérez, & Dewettinck, 2013), and release characteristics. Fat–lecithin mixtures with saturated fatty acids are used as basis for release agents, where the same properties are usefully applied (Dorfman, 2012; Oexmann, 2008). The chain length and degree of saturation provides different adhesion behavior of the fat onto surfaces, which results in different contact angle values (Ashokkumar & Adler‐Nissen, 2011).

Also, water source had an effect on sticking behavior; distilled water reduced take‐off force, although this did not significantly influence the number of sticking waffles. Water of lower water hardness has a lower mineral content (Forghani, Joong‐Hyun, & Deog‐Hwan, 2015). According to Tiefenbacher (2009), the lower the mineral content in batters, the better is usually the release of the waffle and the less batter residue is left on the baking plates. But it has to be considered that waffle stability is also influenced by the mineral content of the batter, it decreases when the mineral content is too low. A balance of mineral content by using water of medium hardness seems to be required (see Fig. 1). Mineral content of the batter is not only a result of the used water but also of the used ingredients, for example, leavening agents are also a source of (different) minerals. In this study, magnesium hydroxide carbonate (see Fig. 1) and ammonium bicarbonate showed lowest number of sticking waffles and lower adherence forces. Magnesium hydroxide carbonate has been experienced to decrease sticking of wafers (Tiefenbacher, 2009) and thus seems to be better suitable for waffles. Ammonium bicarbonate is usually added in characteristic products only (e.g., ginger bread) because of its specific taste.

Take‐off force was negatively correlated with aw‐value, L*‐value, and number of sticking waffles, and positively correlated with a*‐value and baking loss, which themselves were influenced by the batter ingredients. In other words, the required take‐off force decreased when the waffles were brighter and not burnt (increased L*‐value), and when the aw‐value was higher and the baking loss lower, indicating a stable, soft waffle. When a waffle was dark or burnt, also indicated by an increased baking loss and decreased moisture content or water activity, it required higher take‐off force. This can lead to more sticking waffles in the end, although correlation between take‐off force and number of sticking waffles was not significant for these trials.

For predicting the sticking or adhering behavior of waffles during industrial production, determination of moisture content, water activity, baking loss, or color values might be practicable parameters to allow a rough and quick estimation. This was also detected by other researchers, who used color measurements to predict baking state of crackers or moisture content (Broyart, Trystram, & Duquenoy, 1998) and temperature for regulating cake baking quality in tunnel ovens (Baik, Marcotte, & Castaigne, 2000; Sablani, Marcotte, Baik, & Castaigne, 1998; Zareifard, Boissonneault, & Marcotte, 2009).

## 4 Conclusion

The results of this study showed that sticking behavior of waffles was influenced by different types of fats, leavening agents, and water hardness. Ingredients that decreased the number of sticking waffles and their adherence tendency were cocos fat, butter, margarine, ammonium bicarbonate, and magnesium hydroxide carbonate.

For the prediction of adherence or sticking behavior of waffles, the determination of take‐off force, moisture content, water activity, baking loss, and color seemed to be promising and practicable parameters, which can be applied in laboratory as well as in industrial scale. Additionally, the determination of waffle texture is recommendable, as waffle stability was found to have a high influence. Besides these recipe parameters, the baking process is another important factor for waffle production. Contact time and temperature, as well as baking plate material and the type of release agent have to be considered in this respect.

## Funding Information

No funding information provided.

## Conflict of Interest

None declared.

## References

[fsn3425-bib-0001] Ashokkumar, S. , & Adler‐Nissen, J. (2011). Evaluating non‐stick properties of different surface materials for contact frying. Journal of Food Engineering, 105, 537–544.

[fsn3425-bib-0002] Baik, O. D. , Marcotte, M. , & Castaigne, F. (2000). Cake baking in tunnel type multi‐zone industrial ovens Part I. Characterization of baking conditions. Food Research International, 33, 587–598.

[fsn3425-bib-0003] Bringhurst, T. A. & Brosnan, J. (2014). Scotch whisky: Raw material selection and processing In Russell I. & Stewart G. (Eds.), Whiskey technology, production and marketing (2nd edn, Chapter 6, pp. 49–122). Oxford, UK: Elsevier.

[fsn3425-bib-0004] Broyart, B. , Trystram, G. , & Duquenoy, A. (1998). Predicting colour kinetics during cracker baking. Journal of Food Engineering, 35, 351–368.

[fsn3425-bib-0005] Dorfman, M. R . (2012). 19 ‐ Thermal Spray Coatings In Kutz M., Handbook of Environmental Degradation of Materials (Second Edition) (pp. 569–596). Oxford: William Andrew Publishing.

[fsn3425-bib-0006] Forghani, F. , Joong‐Hyun, P. , & Deog‐Hwan, O. (2015). Effect of water hardness on the production and microbicidal efficacy of slightly acidic electrolyzed water. Food Microbiology, 48, 28–34. 25790988 10.1016/j.fm.2014.11.020

[fsn3425-bib-0007] Hadnađev, D. , Hadnađev, M. , Milica, P. , Slađana, R. , & Veljko, K. (2015). Functionality of OSA starch stabilized emulsions as fat replacers in cookies. Journal of Food Engineering, 167, 133–138.

[fsn3425-bib-0008] Hesso, N. , Garnier, C. , Loisel, C. , Chevallier, S. , Bouchet, B. , & Le‐Bail, A. (2015). Formulation effect study on batter and cake microstructure: Correlation with rheology and texture. Food Structure, 5, 31–41.

[fsn3425-bib-0009] Huber, R. , & Schoenlechner, R. (2016). Waffle production: Influence of batter ingredients on sticking of fresh egg waffles at baking plates – Part I: Effect of starch and sugar components. Food Science and Nutrition, 2016, 1–9. 10.1002/fsn3.424 PMC5448360 28572935

[fsn3425-bib-0010] Liu, W. , Christian, G. K. , Zhang, Z. , & Fryer, P. J. (2002). Development and Use of a Micromanipulation Technique for Measuring the Force Required to Disrupt and Remove Fouling Deposits. Food and Bioproducts Processing, 80, 286–291.

[fsn3425-bib-0011] Miller, R. (2016). Leavening agents In Caballero B., Finglas P. M., & Toldra F. (Eds.), Encyclopedia of food and health (pp. 523–528). Oxford, UK: Elsevier, Academic Press.

[fsn3425-bib-0012] Oexmann, T . (2008). Device for the production of wrapped wafer cones for individual wafer, comprises baking molds movably coupled to each other over a stretch of way and having a lower mold and an upper mold. DE102008045832A1 Patent.

[fsn3425-bib-0013] Palav, T. S. (2016). Chemistry of cake manufacturing In Wrigley C. W., Corke H., Seetharaman K., & Faubion J. (Eds.), Encyclopedia of food grains (pp. 367–385). Oxford, UK: Elsevier.

[fsn3425-bib-0014] Pareyt, B. , Talhaoui, F. , Kerckhofs, G. , Brijs, K. , Goesaert, H. , Wevers, M. , & Delcour, J. A. (2009). The role of sugar and fat in sugar‐snap cookies: Structural and textural properties. Journal of Food Engineering, 90, 400–408.

[fsn3425-bib-0015] Sablani, S. S. , Marcotte, M. , Baik, O. D. , & Castaigne, F. (1998). Modeling of Simultaneous Heat and Water Transport in the Baking Process. LWT ‐ Food Science and Technology, 31, 201–209.

[fsn3425-bib-0016] Schünemann, C. , & Treu, G. (2009). Technologie der Backwarenherstellung. Alfeld, Germany: Gildebuchverlag.

[fsn3425-bib-0017] Sciarini, L. S. , Van Bockstaele, F. , Nusantoro, B. , Pérez, G.T. , & Dewettinck, K . (2013). Properties of sugar‐snap cookies as influenced by lauric‐based shortenings. Journal of Cereal Science, 58, 234–240.

[fsn3425-bib-0018] Tiefenbacher, K . (2009). Handbook of wafer technology. In House handbook for Franz Haas Waffel‐ und Keksanlagen‐Industrie GmbH, Leobendorf, Austria, p 1‐C3.

[fsn3425-bib-0019] Tiefenbacher, K. , Haas, F. , & Haas, J . (1991). Process for manufacturing decomposable thin‐walled starch based mouldings. EP0513106B1.

[fsn3425-bib-0020] Vetter, J. L. (2003). Leavening Agents. Encyclopedia of Food Sciences and Nutrition, 2, 3485–3490.

[fsn3425-bib-0021] Zareifard, M. R. , Boissonneault, V. , & Marcotte, M. (2009). Bakery product characteristics as influenced by convection heat flux. Food Research International, 42, 856–864.

